# Carcinoma-like vascular density in atypic keratoacanthoma suggests malignant progression

**DOI:** 10.1038/sj.bjc.6600622

**Published:** 2002-11-12

**Authors:** S Strieth, W Hartschuh, L Pilz, N E Fusenig

**Affiliations:** Division of Differentiation and Carcinogenesis, German Cancer Research Center (DKFZ), Heidelberg, Germany; Central Unit of Biostatistics, German Cancer Research Center (DKFZ), Heidelberg, Germany; Department of Dermatology, University Hospital, Heidelberg, Germany

**Keywords:** angiogenesis, keratoacanthoma, atypia, vascularisation, VEGF

## Abstract

Differential diagnosis between keratoacanthomas and well differentiated squamous cell carcinomas based on clinical and histomorphological data is problematic. Recent findings of cellular atypia in a large proportion of keratoacanthomas indicated that these potentially ‘self-healing’ cutaneous neoplasms had the potential for malignant progression. Another malignancy-associated criterion is enhanced angiogenesis with increased microvessel density. To provide further diagnostic markers for keratoacanthomas we examined microvessel density on paraffin sections of 13 keratoacanthomas in comparison with 10 normal skin biopsies and 16 late-stage skin squamous cell carcinomas by counting and by computer-assisted image analysis of CD31-immunostained vessels. A significant increase of microvessel density in ‘hot spots’ was observed in keratoacanthomas as compared to normal skin. Furthermore, when keratoacanthomas were subdivided into tumours with and without malignancy-associated atypic areas, only those with atypia (*n*=6) were significantly better vascularised than normal skin and had a mean microvessel density in the range of late-stage squamous cell carcinomas. Both keratoacanthoma subtypes revealed comparable levels of inflammatory cell infiltration, tumour cell proliferation and vascular endothelial growth factor expression (mRNA and protein). Thus, in addition to malignancy-associated cellular atypia, increased microvessel density may serve as further diagnostic parameter to discriminate keratoacanthomas with a potential to progress to malignancy.

*British Journal of Cancer* (2002) **87**, 1301–1307. doi:10.1038/sj.bjc.6600622 www.bjcancer.com

© 2002 Cancer Research UK

## 

Keratoacanthoma (KA) is a cutaneous squamous neoplasm arising preferably from hair follicle cells on sun-exposed skin (Schwartz, 1994). First described in 1950 by Rook and Whimster as a rapidly growing but self-healing skin tumour its differential diagnosis and its dignity has been disputed for a long time (Rook and Whimster, 1979). This is also notable in the various nomenclature ranging from ‘tumourlike keratosis’ or ‘self healing epithelioma’ to ‘self healing squamous cell carcinoma’ (for review see Schwartz, 1994). However, in contrast to low grade variants of squamous cell carcinoma (SCC), KAs clinically present with a rapid growth phase for the first 4–8 weeks and a possible spontaneous self-induced regression after 3–6 months. So, clinically, there are three stages of KA: the proliferative stage, the mature/fully-developed stage and the involutional stage (Schwartz, 1994). Due to its similarity in growth rate and morphology to rapidly growing well differentiated SCCs (Billingsley *et al*, 1999) some authors believe it is a well-differentiated variant of cutaneous SCCs (Manstein *et al*, 1998) or at least an ‘abortive malignancy, which only rarely progresses into SCC’ (Schwartz, 1979).

Cells of KAs harbour similar genetic changes as malignant SCCs, for example mutations in the p53-tumour suppressor gene (Kerschmann *et al*, 1994) or an activated ras-oncogene (Corominas *et al*, 1991). Using flow cytometry analysis, neither DNA content nor the proliferative index discriminated SCCs from KAs (Randall *et al*, 1990). But recent studies on the genetics of epithelial skin tumours reveal multiple differences in loss of heterozygosity between KAs and SCCs (Waring *et al*, 1996).

Immunohistochemically there is no definite means to distinguish between SCCs and KAs. Merely different patterns of distribution of involucrin (Smoller *et al*, 1986) and proliferation markers as PCNA (Phillips and Helm, 1993) or Mib-1 (Skalova and Michal, 1995) have been described. Recently, different patterns of immunohistochemical staining of desmoglein-1 and -2 as markers for desmosomes have been observed indicating that a loss of desmosomes is predominantly observed in SCCs and not in KAs (Krunic *et al*, 1998). This is in accordance with earlier ultrastructural studies claiming larger intercellular spaces and a loss of desmosomes in SCCs as a main feature distinguishing KAs from SCCs (Miracco *et al*, 1992).

Nevertheless, differential diagnosis remains critical (Cribier *et al*, 1999) and therefore has to be based on both clinical history and common histologic criteria, especially the lack of invasion into the dermis.

More recently, there have been new data that stress the impact of atypia on clinical presentation and prognosis of KAs (Sanchez *et al*, 2000). The authors even found in about one third of KAs areas with cellular and nuclear atypia indicative of malignant transformation and thus support the hypothesis that those KAs of preferentially sun-exposed locations and diagnosed in older patients may progress to malignant SCCs.

Another hallmark of malignant tumours is the induction of persistent angiogenesis with its consequence of increased vessel density in the tumour stroma. Malignant tumours critically depend on angiogenesis for continued growth, invasion, and formation of metastases (Folkman, 1995). Accordingly, malignant epithelial skin tumours such as SCCs reveal significantly higher microvessel density (MVD) than perilesional skin (Weninger *et al*, 1997). Moreover, in skin SCCs the angiogenic switch occurs not until late-stage tumours penetrate deeper into the dermis and have a higher metastatic risk, so that vascular density appears to be of prognostic significance (Strieth *et al*, 2000). On the other hand, increased MVD has also been detected in KAs (Weninger *et al*, 1997) and in hyperproliferative lesions such as psoriasis (Detmar *et al*, 1994). In all these hyperproliferative stages of the epidermis, including normal wound healing, the vascular endothelial growth factor (VEGF) is considered a major inducer of angiogenesis, because it is overexpressed in those epithelia (Brown *et al*, 1992; Detmar *et al*, 1994; Weninger *et al*, 1996).

We have shown earlier that VEGF expression was similar in premalignant lesions (actinic keratosis) as well as in early- and late-stage malignant skin SCCs, although MVD was significantly elevated in late stage SCCs only (Strieth *et al*, 2000). Strong VEGF expression had been observed both in SCCs and KA as well as in adjacent normal epidermis and was therefore considered not a valid criterion to distinguish malignant from benign epithelial skin tumours (Weninger *et al*, 1996).

In the present study we have analysed MVD in KA with (mKA) and without (nKA) cellular atypia (Sanchez *et al*, 2000). While MVD correlated neither with inflammatory cell infiltration, rate of tumour cell proliferation nor VEGF expression, increased MVD was restricted to mKAs and equalled MVD in late-stage SCCs (Strieth *et al*, 2000) thus supporting malignant progression.

## MATERIALS AND METHODS

### Patients and material

Formalin-fixed, paraffin-embedded specimens from 13 patients with KAs were analysed. These tissue specimens were derived for therapeutic purposes with fully informed consent of patients and were compared with 10 normal skin biopsies of comparable anatomical locations exhibiting no histopathological alterations. All cases were located in sun-exposed areas of the skin predominantly in the face (61.5%).

### Identification of mature-stage KAs

H&E-stained sections of the paraffin-embedded blocks were examined and the diagnosis of mature, non-involuting KA was confirmed by a dermatopathologist not involved in the analysis of vascularisation.

A high degree of differentiation often exhibiting large spinous cells with eosinophilic ‘glassy’ appearance of the cytoplasm, a central keratinous core with overhanging epithelial ‘lips’ resulting in a symmetric tumour architecture at low magnification and essentially a lack of invasion into the subcutis were mandatory for the diagnosis of mature-stage KAs (Schwartz, 1994).

Central keratinisation of KAs was not used to further subdivide into bud-, dome- or berry-shaped subtypes.

There were no special morphologic (e.g. ‘agglomerate’; ‘centrifugum’; ‘giant’; etc.) or syndromic (e.g. Ferguson-Smith-S.- or Muir-Torre-S.-associated) types of KA among the specimens (for review see Schwartz, 1994).

After the completion of vascular density assessment (see Materials and Methods) the group of KA was further subdivided by a pathologist not involved in MVD analysis in (normal) KAs without atypia (nKA, *n*=7) and KAs with cellular and nuclear atypia as a sign of malignant transformation (mKA, *n*=6): mKAs were characterised by the coincidence of atypical cells that can also be found in SCCs and remnants of typical well differentiated KA cells, respectively. In these lesions symmetry of tumour architecture was partially altered. But in contrast to SCCs no frank invasion was detectable on serial sections neither in nKAs nor in mKAs. Other features as actinically damaged elastic fibers or intraepithelial microabscesses were observed in some specimens, but were not included for the diagnosis or classification.

In addition to these histopathological criteria clinical data were used for diagnosis (Schwartz, 1994).

### Endothelial cell identification by CD 31 immunohistochemistry

Sections (5 μm thick) were cut from the retrieved tumour blocks, dewaxed and rehydrated in graded ethanol. Endogenous peroxidase was blocked with 0.3% H_2_O_2_ for 15 min. To unmask hidden epitopes, sections were digested with protease type XXIV (10 mg ml^−1^; Sigma, Deisenhofen, Germany) at 37°C for 15 min. Vessels were visualised by immunostaining with a monoclonal antibody against CD31 (clone JC70/A; Dako, Glostrup, Denmark). Sections were incubated for 6 h (the optimal time) with the primary antibody diluted 1 : 40 in PBS and subsequently developed using a streptavidin-biotin-peroxidase system (Amersham, Braunschweig, Germany). Visualisation of the antibody complex was achieved with a nickel-enhanced diaminobencidin reaction (Hsu and Soban, 1982) resulting in black staining of endothelial cell membranes. Sections were counterstained by a modified Masson-Goldner protocol omitting hematoxylin, as described (Strieth *et al*, 2000).

### Quantitation of vessel density

Sections were screened at a magnification of 100× for areas of highest microvessel density, so-called ‘hot spots’ (Weidner *et al*, 1991) and three of such areas were analysed per section. While focusing on the margins of epithelial tissues, areas next to hair follicles and glands were avoided. At a magnification of 200× the black stained vessels were counted in a square grid defining an area of 0.09 mm^2^ (349×264 μm). All black stained single cells or clusters of cells which were separated from other stained areas were counted as single vessels. Vascular density was defined as the mean vessel count of three areas analysed per section. Results obtained from different sections of the same tumour showed a repetitive pattern (data not shown). Additionally, vessel density was determined by computer-assisted image analysis in the same grid-defined area used for the manual count at a magnification of 200× using a Leica Quantimet 600QWin-Image Analysis System (Leica, Wetzlar, Germany) equipped with a 3CCD camera. The black stained vessels were detected automatically using the *H*ue-*S*aturation-*I*ntensity (HSI) system. In cases where interference was observed with other dark material such as erythrocytes or horn material, this was corrected interactively. Vascular density was calculated as the mean of three measurements per section, and plotted as percentage of stained area (% area).

### Assessment of inflammatory cell infiltrate

On parallel H&E-stained sections infiltration of inflammatory cells was assessed without discriminating white blood cell subtypes. Cell density was screened by a pathologist not involved in determining vessel density and classified as ‘no infiltration’, ‘low degree’, ‘intermediate degree’, and ‘strong degree of infiltration’.

### Determination of proliferative activity

Parallel sections of six KAs without atypia (nKA) and four KAs with atypia (mKAs) were analysed for proliferating cells by immunostaining with the monoclonal antibody Mib-1 (Ki67; Dianova, Hamburg, Germany) after 30 min microwave incubation and using the same immunohistochemical method (DAB-nickel) as for the identification of endothelial cells. The ratio of proliferating to non-proliferating cells in tumour tissue was assessed as described (Kerschmann *et al*, 1994).

### *In situ* hybridisation of VEGF-RNA

Human cDNA (649 bp) encoding human VEGF_165_ in Bluescipt KS^−^ vector (Stragagene, La Jolla, CA, USA) (Keck *et al*, 1989), generously provided by Drs H Weich and D Marmé (Freiburg, Germany) was used as a probe. *In situ* hybridisation was essentially performed as described (Moorman *et al*, 1993). In brief, ^35^S-labelled RNA probes for VEGF were prepared using T3 and T7 RNA-polymerase (for antisense and sense probes, respectively) according to the manufacturer's instructions (Roche Diagnostics, Mannheim, Germany). Paraffin sections of normal skin (*n*=3) and KAs (two nKA and one mKA), parallel to those used for determining vascular density, were pretreated, hybridised and washed at high stringency as described (Strieth *et al*, 2000). For autoradiography, slides were coated with NTB2 film emulsion and exposed for 4 weeks. After the film was developed, sections were counterstained with hematoxylin.

### VEGF immunohistochemistry

Indirect immunohistochemistry using a polyclonal antibody against VEGF_165_ (1 : 20; Cat.No. GF 25; Calbiochem, San Diego, CA, USA) was performed on sections of both KA subtypes parallel to those specimens used for *in situ* hybridisation and on additional sections of nKA and mKA cases. Pretreatment of sections was performed by a 30 min incubation in 0.05% Saponin (Sigma, Deisenhofen, Germany). Background staining was blocked by incubating the slides 15 min in streptavidin (1 μg ml^−1^; Sigma, Deisenhofen, Germany) and normal goat serum (1 : 10; Dianova, Hamburg, Germany) before application of the primary antibody. The secondary antibody was diluted in PBS with 5% human serum (Dianova, Hamburg, Germany). To test the specificity of antibody staining, recombinant VEGF_165_ (*E. coli*), kindly provided by Dr D Marmé (Freiburg, Germany), was used for neutralising the antibody as negative control (ligand: antibody=9.7 : 1, based on protein mass) and led to complete abrogation of the antibody reactivity. Evaluation of RNA and protein expression of VEGF was based both on area and intensity of the staining.

### Statistical analysis

The mean vascular area percentage and the mean manual vessel count data have been calculated with the MS Excel software. Data are presented as boxplots or are given as mean±s.d. and have been further analysed by the statistics software Sigmastat (SPSS Inc., Chicago, IL, USA). Linear regression and calculation of Spearman's correlation coefficient was performed to test correlations between different vascular density parameters, inflammatory scores and proliferative rates. The Mann-Whitney *U*-test was used to test differences in vascular density values (% area) between normal skin and KAs and differences in age of nKA and mKA patients, respectively. In addition, vascular density values of nKAs and mKAs were compared to those of normal skin and late-stage SCCs reported earlier (Strieth *et al*, 2000) using the Kruskal-Wallis one way analysis of variance on ranks and Dunn's all pairwise multiple comparison procedure as *post hoc* analysis. *P* values less than 0.05 were considered significant.

## RESULTS

### Clinical data analysis of KA patients

**Figure 1:**
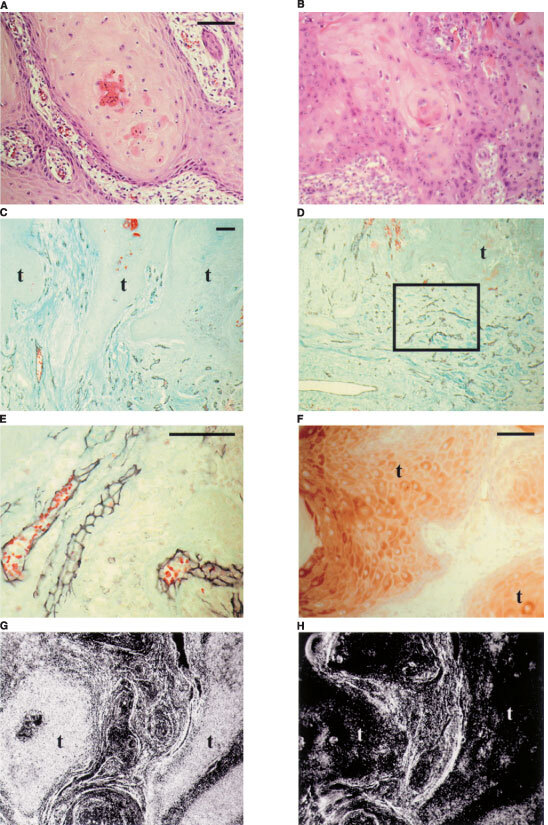
Two subtypes of keratoacanthoma: (**A**) nKA revealing neither cellular nor nuclear atypia (H&E). (**B**) mKA showing prominent cellular and nuclear atypia of tumour cells (H&E). Immunohistochemical staining of microvessels using CD31-antibodies in KA. (**C**) nKA (counterstaining modified Masson-Goldner). (**D**) mKA, ‘hot spot’ in frame (t: tumour). (**E**) Endothelial staining in detail. VEGF expression: (**F**) VEGF protein localisation by immunohistochemistry in KA revealed a distribution with preference to differentiating cells. (**G**) Expression of VEGF-RNA revealed by radioactive *in situ* hybridisation in KA. VEGF anti-sense probe (dark field). (**H**) Corresponding sense probe control (dark field). Bar: 30 μm.

Mature stage KAs were diagnosed according to clinical history and common histopathological criteria as described above (Figure 1A,B

). Nearly all lesions were located in sun-exposed skin, most of them (*n*=8; 61.5%) in the head and neck region, two on arms, two on legs and one on the back. Localisations of the lesions had no significant influence on their vessel density (data not shown). All were primary lesions and no basic disease (e.g. diabetes mellitus or psoriasis) was evident in KA patients which might have affected the degree of vascularisation.

Evaluation of durations of the lesions in nKA patients, as compared to mKA patients was difficult because these clinical data were only available in less than 50% of the cases. Therefore no statistical analysis was performed. A tendency to increased durations in mKA patients (nKA: 0.9±0.4 months *vs* mKA: 3.2±2.0 months) must not be ovestimated because it was essentially due to *one* mKA lesion noticed by the patient for 6 months before diagnosis.

### Immunohistochemical detection of blood vessels

Reaction of antibodies against CD31 provided an uniform and intense membrane staining of endothelial cells lining smaller and larger vessels with no or very low background. Using the nickel enhancement procedure endothelial cells forming the walls of vessels of all sizes were easily identified and even very small vessels clearly recognised (Figure 1E). The marked vessels were counted and the density of the stained area morphometrically assessed in three framed areas per section choosing ‘hot spots’ of vascular density. In control sections of anatomically related normal skin, a typical capillary bed of normal, uninflamed and non-irritated dermis was visible. In areas adjacent to tumour cell nests vascularisation of KAs appeared clearly increased compared to normal skin (Figure 1D).

### Quantitative assessment of vascularisation in KAs

**Figure 2:**
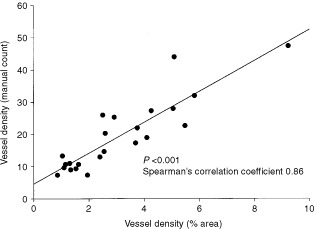
Linear regression of the two vascular density parameters: manual count *vs* % area.

**Figure 3:**
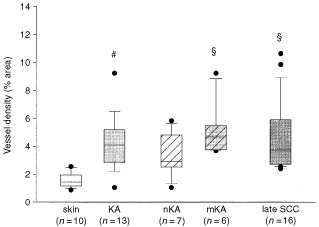
Vessel density in normal skin and KA and the subtypes of KA: nKA, mKA compared to late-stage SCCs (# *P*<0.001 significant *vs* skin, § *P*<0.05 significant *vs* skin using all pairwise multiple comparison).

In order to quantitate vascularisation of KAs, MVD was assessed in tumour stroma both by manual count and computer-assisted morphometric analysis of stained areas in at least three regions of interest on each tumour section. When comparing both quantitation methods in tumour and normal skin sections, respectively, the data were very well correlated (Spearman's correlation coefficient: 0.86; Figure 2

). For comparison with normal skin and SCC, respectively, data obtained by morphometrical analysis of stained vessel area were used. When compared to normal skin, vascular density in the tumour-adjacent stroma of all KAs was more than twice higher and this was highly significant (*P*<0.001; Figure 3

).

However, upon subdividing the group of KAs (*n*=13) in KAs without atypia (nKAs, *n*=7, Figure 1C) und KAs with atypia (mKAs, *n*=6, Figure 1D) only mKAs were significantly better vascularised than normal skin (*P*<0.05; Figure 3).

In order to directly compare SCCs, nKA and mKAs, late-stage SCC vascular density values obtained from an earlier study are depicted in Figure 3 (Strieth *et al*, 2000). Considering the present debate of a potential malignant transformation of KA with atypia into an invasive tumour it was intriguing that vascular density in mKAs – but not nKAs – was comparable or even higher than in invasive late stage SCCs (Figure 3). Although nKAs still exhibited increased vascularisation, as compared to normal skin, this was no longer significant and the values were lower than in late-stage SCCs.

### Impact of chronic inflammatory cell infiltrates and tumour cell proliferation

Changes in vascular density might have been caused by inflammatory reactions in the tumour stroma or simply associated with higher proliferation rates of the tumour cells. Therefore, the extent of inflammatory cell infiltration was assessed in H&E-stained sections at a magnification of 200× using an arbitrary scale for the frequency of white blood cells, mainly lymphocytes. While inflammatory cells were absent in normal skin specimens, they were frequent in KAs. Three tumours (one nKA and two mKAs) revealed a ‘low degree’ of inflammatory cell infiltrate, while the majority of KAs (four nKAs and four mKAs) showed an ‘intermediate degree’ of inflammation. Only two tumours, and these were both nKAs, exhibited an inflammatory response considered as ‘strong’. Summarised, there is neither a correlation between these inflammatory cell infiltrates and vascular density values in individual KA lesions (*P*=0.24) nor in the groups of nKAs (*P*=0.92) and mKAs (*P*=0.25), respectively. This documented that the different vascular density values in KAs were not significantly affected by inflammatory cell infiltrates.

The rate of proliferating (Mib-1 positive) epithelial cells was determined in 10 tumours (six nKAs and four mKAs) and seven control specimens of normal skin. All KAs revealed high proliferation rates (48±8%) as compared to normal skin (<10%), but there was no difference observed between nKAs (49±4%) and mKAs (47±12%), respectively. Also, individual tumours with highest vascular density values did not exhibit the highest proliferative activity and both parameters were not correlated (*P*=0.65). Thus, neither inflammatory response in the stroma nor the proliferation rates of tumour cells were obviously responsible for the increased vascular density observed in mKAs.

### Expression of VEGF-RNA and -protein

In order to evaluate whether elevated levels of expression of a major angiogenesis inducing factor, i.e. VEGF, might have caused the increased vascularisation in KAs, mRNA expression and protein levels of VEGF were analysed in parallel sections.

By radioactive *in situ* hybridisation strong VEGF expression in epithelial tumour cells was detected in both nKA and mKA (one nKA, two mKAs; Figure 1G,H). In epidermis of normal skin, respectively, the expression was also apparent but weaker (data not shown). There was no significant intertumoural variability of the labelling. Label was observed in all epithelial cell layers, though with a tendency to more intensity in differentiated cell layers. The sense probe only yielded some background labelling (Figure 1H).

In addition, VEGF-protein was identified by indirect immunohistochemistry in parallel sections of the same KAs and on additional sections from each group (nKA: *n*=2 and mKA: *n*=4). There was no obvious difference in staining intensity between nKAs and mKAs and the staining pattern in all tumours was very homogenous again, with higher intensity in differentiated cells (Figure 1F). In normal skin the protein staining was very weak.

As a control for the specificity of the antibody, the immunoreaction was abolished following blocking of the antibody with recombinant human VEGF.

When compared to VEGF-expression (RNA- and protein level) observed in human skin SCCs as reported earlier (Strieth *et al*, 2000), there were no apparent quantitative differences in intensity or localisation identified to that assessed in KAs.

The data obtained for KA in this study further confirm that there is neither an obvious association between the expression of VEGF and atypia in the tumours nor between VEGF expression and intratumoural vascularisation.

## DISCUSSION

Tumour growth and metastasis are dependent on persistent new blood vessel growth (Folkman, 1995), and intratumoural MVD is a significant prognostic indicator of tumour progression (for review see Weidner, 1995). With regard to skin carcinogenesis when premalignant lesions and early as well as late-stage (according to Breuninger *et al*, 1990) SCCs were analysed, significantly increased MVD, as compared to normal skin, was only observed in late-stage SCCs (Strieth *et al*, 2000). This suggests that increased vascularisation may have prognostic significance for the progression of these tumours.

Accordingly, angiogenesis and MVD in KAs are of particular interest in view of the heterogeneity of these neoplasms, with a subgroup resembling initial SCCs (Sanchez *et al*, 2000). Here we provide new evidence that changes in MVD, in addition to histologic atypia, discriminate KAs into two subgroups with different potential of progression to malignancy. This may have prognostic value for this critical skin lesion.

The relationship between KA and squamous cell carcinoma has been debated since the concept of KA was proposed in 1950 by Rook and Whimster (Rook and Whimster, 1979). It is still a hypothetical question whether KA is a distinct ‘pseudomalignancy’ or a form of SCC that spontaneously involutes in the majority of cases but rarely metastasises. So far, there is lack of evidence that involution of KAs really occurs or at least at what percentage KAs involute, if left alone (Leboit, 2002). There are even reports about intravascular and perineural invasion or even lymph node metastasis (for review see Hodak *et al*, 1993) Furthermore, tumour regression is reported for typically malignant tumours of the skin as well (Barnetson and Halliday, 1997). On the other hand, it is not clear yet whether and how frequently malignant progression of KAs to SCCs occurs. Reed (1972) was the first to raise this question based on his observation that 29% of KAs in his files had strong morphologic similarities to SCCs, representing KAs with a degree of nuclear atypia indistinguishable from that seen in SCC. Due to the lack of clear diagnostic and prognostic criteria to distinguish between KA and SCC, all those tumours are currently surgically excised (Rinker *et al*, 2001).

Regarding the dignity of KA the best criterion appears to be atypia (Leboit, 2002). While KA cells with no discernibly atypical nuclei are associated with a possibly involuting lesion, aggregations of cells with anaplastic appearance are found in mKA as well as in SCCs and thus favour malignant progression.

Recently, in a retrospective study revising 220 crateriform proliferative squamous cell lesions new evidence was found that KA might progress into SCC (Sanchez *et al*, 2000). While histologically 29 of these cases turned out to be crateriform SCCs, 144 were diagnosed KA without atypia (nKA), but in 47 cases there were signs of frank histologic and cytologic atypias indicative of malignant transformation. In addition, the authors found that patients with nKA were significantly younger than patients with mKA or those with SCCs. Moreover, patient history revealed reduced durations of the lesions in patients with nKA, as compared to mKA. Therefore, based on these histologic *and* clinical features, the subtype of atypical KA with probability to malignant conversion (mKA) was proposed. In the present study, comprising a rather small number of KAs, it was surprising that about half of the tumours showed signs of atypia.

An experimental approach to address the question whether SCCs develop within KAs was attempted in transgenic rabbits using cottontail-rabbit-papillomavirus-targeted gene expression of the EJ*ras* oncogene to epidermal keratinocytes (Peng *et al*, 1999). One hundred per cent of F1 animals developed KAs on day 3 after birth and 18% developed SCCs 5 months later. Although the authors did not find direct evidence so far for malignant conversion of KAs into SCCs, the sequential induction of both tumour types by the same oncogene suggested common transformation mechanisms. In addition, there are further reports of spontaneous skin tumour development with histological features both of KA and SCCs in ageing Long Evans rats (Esfandiari *et al*, 2002).

The consideration of atypia as a sign of malignant progression in skin tumours is not restricted to KAs: For example, there were heterogenous reports about the presence of atypical CD30^+^ cells in lymphomatoid papulosis – another potentially ‘self-healing’ cutaneous pseudomalignancy (el-Azhary *et al*, 1994). For this typically waxing and waning skin lesion it is well documented that malignant progression occurs (Drews *et al*, 2000). It has further been shown that this skin neoplasm definitely is a monoclonal T-cell disorder of atypical CD30^+^ cells (Steinhoff *et al*, 2002) and that these atypical cells are even functionally linked to malignant progression (Levi *et al*, 2000).

There are other examples among skin ‘pseudomalignancies’ where atypia is now associated with a potential to progress, for example atypical fibroxanthoma (now: superficial malignant histiocytoma), regressing atypical histiocytosis (now: anaplastic large cell lymphoma) and proliferating pilar tumour (regarded as form of SCC) (Leboit, 2002).

The correlation of an angiogenic phenotype and other hallmarks of biologic aggressiveness in tumours of the skin is well accepted: For example, basal cell carcinomas (BCC) showing a higher vascular density appeared to be more aggressive in terms of deeper local infiltration or formation of distant metastasis compared to low-vascularised relatively benign BCCs (Staibano *et al*, 1996). Moreover, it has been found that the angiogenic response discriminates between cutaneous B-cell lymphomas and B-cell pseudolymphomas with the malignant variant exhiting increased MVD (Schaerer *et al*, 2000). Weninger *et al* (1997) investigated MVD in BCCs, SCCs and KAs. While they found a significantly lower vascularisation in BCCs there was no significant difference in MVD between SCCs and KAs. However, in this study, no discrimination was made between KAs without or with atypia.

In accordance with the data reported by Weninger *et al* (1997) we found that KAs are strongly vascularised tumours. By further subdividing KAs into lesions with and without atypia we observed that only mKAs (with atypia) were equally well or even better vascularised than late-stage SCCs. On the other hand, KAs without atypia (nKAs) were less vascularised than SCCs and did not significantly differ from normal skin. This difference in MVD was not influenced by histological stages of KA development, because only mature, i.e. non-involuting lesions had been examined. This is also evident by the high proliferative activity of tumour cells and excludes the possibility that involuting KAs with their decreased angiogenesis and vascular density would have obscured the data.

The expression of VEGF was high in both KA subtypes indicating that this angiogenic factor did not play a decisive role. Strong VEGF expression was similarly detected in SCCs and even in premalignant actinic keratosis, although vascularisation was significantly different in both lesions (Strieth *et al*, 2000). In general, VEGF is rapidly up-regulated in different epidermal lesions such as wound healing, common warts, seborrheic keratoses, skin neoplasias (including KAs) and also detected in normal epidermis (Weninger *et al*, 1996). The role of VEGF receptors in skin angiogenesis is less clear though. Heterotransplant studies of benign and malignant skin keratinocytes on nude mice had demonstrated that the expression of both VEGFR-1 and -2 paralleled induction of tumour angiogenesis, and it was coincidentally down-regulated with decreasing vascularisation (Skobe *et al*, 1997; and unpublished observations).

Other controlling factors or conditions which might play a role in tumour angiogenesis in KAs cannot be ruled out. Inflammatory cell infiltrates, as less specific angiogenesis inducers and frequently observed in epithelial skin neoplasms (Coussens *et al*, 1999), differed neither in distinct stages of skin carcinogenesis from actinic keratosis to SCCs (Strieth *et al*, 2000) nor in the KA subtypes, and are thus less likely to be involved in the enhanced angiogenesis in KAs.

In summary, the data presented in this study show a strong correlation between occurrence of atypia and increased vascular density in KAs. Although this has to be substantiated in a large number of tumours, vascular density might be an additional prognostic factor indicating the probability of malignant progression of KA into SCC.
